# Oxidative profile of sickle cell patients in a Cameroonian urban hospital

**DOI:** 10.1186/s12907-016-0037-5

**Published:** 2016-09-21

**Authors:** Vicky Jocelyne Ama Moor, Constant Anatole Pieme, Bernard Chetcha Chemegne, Helene Manonji, Borgia Legrand Njinkio Nono, Corine Tchoula Mamiafo, Bruno Moukette Moukette, Francine Tankeu Nzufo, Asonganyi Tazoacha

**Affiliations:** 1 Department of Biochemistry and Physiological Sciences, Faculty of Medicine and Biomedical Sciences, University of Yaoundé I, PO Box 1364, Yaounde, Cameroon; 2 Laboratory of Biochemistry, University Teaching Hospital, Yaounde, Cameroon; 3 Departement of Haematology, Faculty of Medicine and Biomedical Sciences, University of Yaoundé I, PO Box 1364, Yaounde, Cameroon; 4 Heamatology Unit, Central Hospital, Yaounde, Cameroon; 5 Department of Pharmacy and Traditional Medicine, Faculty of Medicine and Biomedical Sciences, University of Yaoundé I, PO Box 1364, Yaounde, Cameroon

**Keywords:** Sickle cell disease, Oxidative stress, Anti-oxidants, Drépanocytose, Le stress oxydatif, Anti-oxydants

## Abstract

**Background:**

Sickle cell disease (SCD) is a class of hemoglobinopathy resulting from a single mutation in the ß-globin chain inducing the substitution of valine for glutamic acid at the sixth amino acid position which leads to the production of abnormal haemoglobin (haemoglobin S [HbS]). Studies demonstrated the implication of oxidative stress in the development of the sickle cell disease.

**Methods:**

The study aim was to determine the level of oxidative stress markers in a group of sickle cell homozygous patients (SS) in the Yaounde Central Hospital above 15 years of age. Hemolysates obtained from patients were used to investigate some oxidative stress markers including malondialdehyde (MDA), nitric oxide (NO), catalase (CAT), superoxide dismutase (SOD), peroxidase, total antioxidant capacity (TAC) and total protein concentration.

**Results:**

Eighty four individuals, 42 males and 42 females participated (50 % each) with an age range of 15 to 55 years. The levels of markers were significantly higher in the healthy AA group than sickle (SS) (*p* < 0.05), with the exception of MDA which was significantly high in sickle cell (SS) patients than healthy (*p* = 0.037). With respect to the gender, both healthy and SS females showed a greater Total anti-oxidant capacity (65 μM) compared to the males (55 μM).

**Conclusion:**

The increase in the oxidative stress level especially MDA in sickle cell homozygous patients compared to healthy AA individuals confirms that oxidative stress is involved in the pathogenesis of the sickle cell disease.

## Background

Sickle cell disease (SCD) was the first disease to be characterized by a mutation of a gene encoding the human β-globin subunit with the resulting replacement of β6 glutamic acid by valine [1]. Sickled Hb is associated with steady state increases in plasma cell-free hemoglobin and overproduction of reactive oxygen species (ROS). The generation of reactive oxygen species is a steady-state cellular event in respiring cells and their production can be amplified in response to a variety of pathophysiological conditions such as inflammation, immunologic disorders, hypoxia [2]. Reactive oxygen species can cause damage to biological macromolecules such as proteins, lipids and DNA. Patients with SCD are subjected to increased oxidative stress particularly during vaso-occlusive crises and acute chest pain [1, 3]. Oxidative stress represents the imbalance between enhanced generation of reactive oxygen species and low cellular content of antioxidants. Oxidative damage has been shown to change a number of RBC membrane properties, alter membrane permeability and lead to hemolysis. The major defense systems against ROS and oxidative stress include those that scavenge free radicals such as glutathione, vitamin C, vitamin E and superoxide dismutase and that reduce hydroperoxides formed by glutathione peroxidase and catalase.

Sickle-cell anemia has a high prevalence throughout equatorial Africa; additionally, the genetic defect is now known to be widespread in parts of Sicily and southern Italy, northern Greece, southern Turkey, the Middle East, Saudi Arabia, much of central India, and the Americas [4]. The prevalence of sickle cell trait in western, central and eastern Africa varies from 5 to 40 % but it is less common in northern and southern Africa [5]. Three quarters of all sickle cell cases occur in Africa and the total number of carriers in the world is estimated to be about 120 million. In Cameroon, the prevalence of the sickle cell trait is estimated to be 18.2 % for the heterozygous form and 2–3 % for the homozygous SS forms [3]. The prevalence of SCD is increasing and becoming alarmingly common in the Cameroon. Very few studies have been carried out on the role of free radicals and profile of anti-oxidant in this region. Moreover no study has been conducted to compare pro-oxidant and antioxidant status of homologous patients. It is relevant therefore to evaluate the oxidative status of homozygous SS patients and to identify whether these markers could be associated with the physiopathology of the disease.

## Methods

### Recruitment of participants

A total of eighty four patients were included (42 sickle cell patients and a control group of 42 healthy individuals matched for age and sex with the patients) in the study. They were all above 15 years of age. The study was carried out in the Yaounde Central Hospital. Analytical study involved the comparison of two groups. The study took place from December 2013 to April 2014. The sample size was calculated using the Formula of Lorentz which takes into consideration the prevalence of homozygous SS patients in Cameroon. (2–3 %) [6]. None of the patients or the controls had a history of concomitant chronic illness like diabetes, evidence of cardiovascular, hepatic, renal, or gastrointestinal disease or the presence of chronic infectious illnesses (Rhumatoid athritis, AIDS) and had not received a blood transfusion within 3 months of the study. Information on the study was given to the potential participants and their legal guardians (if need be) in their first official languages. Patients read and signed the informed consent form. For each participant, demographic data was obtained and noted unto a pre structured data collection sheet. The study was approved by the ethical committee of the Faculty of Medicine and Biomedical Sciences of the University of Yaounde 1 (Cameroon).

### Blood sample collection

Five milliliters of venous blood was collected from each participant into a tube containing Ethylene Diamine Tetra Acetate (EDTA). The blood was then centrifuged at 1000 rpm for 15 min. The plasma and buffy coat were removed and sickle RBCs were lysed with ice cold water and the clear lysate obtained after spinning down the cell debris at 5000 rpm for 10 min at 4 °C was used for the assays.

### Biochemical analyses

The superoxide dismutase (SOD) activity was determined using the Misra and Fridovich method which is based on the fact that SOD inhibits the oxidation of adrenaline to adrenochrome. Adenochrome formed can be detected in a spectrophotometer at 480 nm by its pink color [7].

The catalase activity was determined following the addition of hydrogen peroxidase and dichromate/acetic acid by the detection of perchromic acid precipitates whose absorbance was read at 570 nm by spectrophotometry [8]. The catalase activity was expressed as micromoles of H_2_O_2_/ min/mg of protein.

Lipid peroxidation assay was performed by a formerly described protocol [9]. Aldehydes, lipid peroxidation of products and malondialdehyde (MDA), an end product of lipid peroxidation of erythrocytes, can react with thiobarbituric acid (TBA) to form a colored complex called thiobarbituric acid reactive substance (TBARS), which was assayed spectrophotometrically at 532 nm and expressed in micro moles.

The Nitric Oxide (NO) determination was done by using the Greiss’ reagent. In this experiment, nitrite is mixed with sulphanilamide and then with N-naphthyl ethylenediamine. This produces a purple colour and the concentration of nitric acid can be read at an absorbance of 540 nm and expressed in micro molar [10].

Peroxidase activity was determined by the peroxidase kit (CAS Number 7722-84-1, Sigma Aldrich). It as an enzymatic method using pyrogallol as the substrate to determine the activity of peroxidase and the absorbance was read at 420 nm.

The total antioxidant capacity (TAC) of the hemolysate was measured using a Ferric Reducing Antioxidant Power (FRAP), a method that determined the capacity of a sample to reduce iron (Fe^3+^) at the acidic pH (3.6). An intense blue color is formed when ferric tripyridyltriazide (TPTZ-Fe^3+^) complex is reduced to ferrous tripyridyltriazine (TPTZ-Fe-^2+^) and the absorbance measured at 593 nm. The result is expressed in micro molar [11].

### Statistical analyses

The results were presented as mean ± standard deviation using the software Graphpad Insat. The non-parametric Mann Whitney U test was applied to detect the significant difference between the groups. We also used ANOVA for one way analyses. Non-parametric distribution was analysed by Kruskal Wallis followed by Dunn’s multiple Comparison test which is a post – hoc test with a *p*- value of < 0.05 considered statistically significant.

## Results

Eighty four patients were included in the study among them 42 sickle cell patients and 42 healthy individuals matched for age and sex (Table 1). There was an equal distribution of males and females in each group, giving a male to female ratio of 1:1. Our target age for sickle patients was 15 to 55 years old with a mean of 25 ± 8 years. Concerning our control group, the age range was from 18 to 53 years with a mean age was 26 ± 7 years. There was a general decrease in the Body Mass Index (BMI) of sickle cell patients as compared to healthy individuals in both male and female groups (Table 1). The levels of SOD, CAT, TAC and NO were significantly (*p* < 0.05) higher in the healthy group compared to the sickle cell group. On the contrary, the concentration of MDA was significantly higher in the sickle group. There was no significant difference in the peroxidase activity of both groups (Table 2). Tables 3 and 4 showed the variation of oxidative marker with respect of sex for sickle and healthy patients. The results showed that except for TAC which was significantly higher in the sickle female patients group, there was not significant variation in the markers between males and females. The concentration of TAC was also significantly higher in females in the AA controls patients (Table 4).

**Table 1:** Age and body mass index (BMI) of participants

Gender	Age (years) (mean ± δ)	BMI (kg/m^2^) (mean ± δ)
Males SS	26.2 ± 9.0	21.5 ± 3.9
Females SS	24.4 ± 8.6	21.9 ± 3.6
Males control	28.5 ± 8.8	23.4 ± 4.0
Females control	24.4 ± 7.3	25.5 ± 3.3

**Table 2:** Levels of oxidative markers in sickle and healthy individuals

	Catalase (U/mg prot)	SOD (U/mg of prot)	MDA (μM)	Peroxidase (U/mg of prot)	NO (μMl)	TAC (μM)	(MDA*100/TAC)
Healthy AA	795 ± 15	14.9 ± 1.7	0.012 ± 0.002	0.024 ± 0.004	9.8 ± 0.66	0.76 ± 0.07	1.5
Sickle SS	697 ± 20	6.5 ± 1.4	0.018 ± 0.004	0.033 ± 0.006	8.7 ± 0.69	0.68 ± 0.14	2.64
*p*- value	0.0001**	0.002**	0.037**	0.339	0.04**	0.001**	

*AA* healthy patient without sickle cell disease, *SOD* super oxide dismutase, *MDA* malondialdehyde, *NO* nitrogen oxide, *TAC* total antioxydant capacity, *prot* protein

**Statistically significant

**Table 3:** Variation of stress markers in sickle patients with respect to sex

	Catalase (U/mg prot)	SOD (U/mg prot)	MDA (μM)	Peroxidase (U/mg prot)	NO (μM)	TAC (μM)	MDA*100/TAC
SS males	5.2 ± 1.2	0.7 ± 0.15	2.2 ± 0.33	0.03 ± 0.00	8.8 ± 1.5	0.55 ± 0.05	4
SS females	6.5 ± 1.3	0.8 ± 0.11	2.1 ± 0.21	0.05 ± 0.00	8.9 ± 1.8	0.65 ± 0.04	3
*p*- value	0.85	0.163	0.18	0.267	0.50	0.009**	

*SOD* super oxide dismutase, *MDA* malondialdehyde, *NO* nitrogen oxide, *TAC* total antioxydant capacity, *prot* protein

**Statistically significant

**Table 4:** Variation of stress markers in healthy individuals with respect to sex

	Catalase (U/mg prot)	SOD (U/mg prot)	MDA (μM)	Peroxidase (U/mg prot)	NO (μM)	TAC (μM)	MDA^a^100/TAC
Healthy males	5.4 ± 1.2	1.4 ± 0.08	1.7 ± 0.07	0.039 ± 0.00	8.96 ± 0.8	0.59 ± 0.07	2.88
Healthy females	6.5 ± 0.08	1.6 ± 0.1	1.6 ± 0.05	0.04 ± 0.00	8.99 ± 0.4	0.70 ± 0.08	2.2
*p*- value	0.71	0.825	0.187	0.12	0.139	0.00612**	

*SOD* super oxide dismutase, *MDA* malondialdehyde, *NO* nitrogen oxide, *TAC* total antioxydant capacity, *prot* protein

**Statistically significant

## Discussion

The role of oxidant damage to red cells in sickle cell anemia has been of interest in recent years. The generation of reactive oxygen species is a steady-state cellular event in respiring cells. Their production can be grossly amplified in response to a variety of pathophysiological conditions such as inflammation, immunologic disorders, hypoxia, hyperoxia, metabolism of drugs or alcohol, exposure to UV or therapeutic radiation, and deficiency in antioxidants enzymes [12]. SCD is a hereditary disorder with higher potential for oxidative damage due to chronic redox imbalance in red cells that often results in clinical manifestation of mild-to severe hemolysis in patients with this genetic disorder [13]. It was shown that SS patients produced greater quantities of O^2−^, H_2_O_2_ and OH^·^ than AA patients with normal red blood cells [14]. The present study investigated the variation between pro and antioxidant markers of the homozygote sickle and healthy patients and the results showed that the activities of SOD, CAT, NO and TAC were significantly decreased in the SS subjects as compared with the control normal subject group AA. The deficiency of the activities of these enzymes may be attributed to the high production of ROS in these patients which may destroy these antioxidant enzymes [2]. Some previous studies demonstrated that the activities of SOD, CAT and peroxidase were reduced while others reported that the activities of both SOD and peroxidase were increased [15, 16]. The decrease of the levels of SOD in the sickle patients as found in this study could be able to increase the flux of superoxide ion exposing the sickle erythrocytes to high level of hydrogen peroxide. Furthermore, the increase of MDA in the same group can be attributed to the auto-oxidation of iron seen in these patients [15]. Also, the excess production of MDA has additional toxic effects leading to alterations of the proteins, modifications of amino-acid side chain, and lipids structure. These alterations may result in a partial or complete loss of protein functionality including antioxidant enzymes, protein receptors [2, 17] and cause externalization of phosphatidylserine in red cell membranes which can enhance complement activation and cell lysis [12].

The data showed that except for MDA, there was an increase of the remaining oxidative stress markers tested in both females SS and healthy patients. Difference between males and females in this study may also be due to the fact that women have a source of antioxidant protection (oestrogen) which is low or absent in men [18]. Previous study demonstrated similar results [19]. MDA, which is major aldehyde product of lipid peroxidation, reflects damage to lipids. Many studies support the role of estrogens in the primary and secondary prevention of Cardiovascular Disease (CVD) among women, particularly in normalizing blood lipids or inducing endothelium-dependent vasodilation stimulating nitric oxide synthetase [20]. The severe alteration of the oxidative pattern in the male homozygote SS may offer one possible pathogenetic explanation for the higher incidence of crisis and complications observed in SS males than females.

## Conclusion

These results show a statistically significant increase of the concentration of MDA and a statistically significant decrease of catalase, SOD, NO, TAC in sickle cell patients compared to the healthy controls group. The level of peroxidase was not different between the two groups. With respect to the gender, healthy and SS female groups show a greater level of TAC compared to the males. In the sickle cell patients, the reduction of antioxidant defense mechanisms can lead to damage of cellular organelles and enzymes and cause an increase of lipid peroxidation.

## Abbreviations

BMI: Body mass index
CAT: Catalase
CVD: Cardiovascular disease
EDTA: Ethylene Diamine Tetra Acetate
FRAP: Ferric Reducing Antioxidant Power
MDA: Malondialdehyde
NO: Nitric oxide
RBC: Red blood cells
ROS: Reactive oxygen species
SCD: Sickle cell disease
SOD: Superoxide dismutase
TAC: Total antioxidant capacity
TBA: Thiobarbituric acid
TBARS: Thiobarbituric acid reactive substance
TPTZ: 2,4,6-Tri(2-pyridyl)-s-triazine
UV: Ultraviolet

## References

[1] Galactéros F. Bases physiopathologiques de la drépanocytose, prise en charge et actualités thérapeutiques. Bull Soc Pathol Exot. 2001;94:77–9. 11475031

[2] Alsultan AI, Sultan L, Seif MA, et al. Relationship between oxidative stress, ferritin and insulin resistance in sickle cell disease. Eur Rev Med Pharmacol Sci. 2010;14:527–38. 20712260

[3] Klings ES, Farber HW (2001). Role of free radicals in the pathogenesis of acute chest syndrome in sickle cell disease. Respir Res.

[4] Hyacinth I, Oluwatoyosi A, Yilgwan CS (2013). Malnutrition in sickle cell anemia: implications for infection, growth, and maturation. J Soc Behav Health Sci.

[5] Diallo D, Tchernia G (2002). Sickle cell disease in Africa. Curr Opin Hematol.

[6] WHO. Regional Comittee for Africa. Malabo, Equatorial guinea. 30 August- 3 September 2010.

[7] Misra H, Fridovich I (1972). Estimation of superoxide dismutase. J Biochem.

[8] Sinha A (1972). Colorimetric assay of catalase. Anal Biochem.

[9] Gutteridge JMC, Stocks J, Dormandy TL (1974). Thiobarbituric acid-reactive substances derived from autoxiding linoleic and linolenic acids. Anal Chim Acta.

[10] Mathew G, Glenda J, Jack L (1996). Quantitation of nitrite and nitrate in extracellular fluids. Methods Enzymol.

[11] Benzie F, Strain J (1996). The Ferric Reducing Ability of Plasma (FRAP) as a measure of “antioxidant power”: the FRAP assay. Anal Biochem.

[12] Vanusa M, Luisa LL, Ana PS, et al. Blood antioxidant parameters in sickle cell anemia patients in steady state. Original communications. J Nat Med Ass. 2008;100(8):897–902. 10.1016/s0027-9684(15)31402-4 18717139

[13] Chan AC, Chow CK, Chiu D (1999). Interaction of antioxidants and their implication in genetic anaemia. Proc Soc Exp Biol Med.

[14] Nanfack P, Biapa Nya PC, Pieme CA, Ama Moor VJ, Moukette B, Ngogang YJ (2013). The in vitro antisickling and antioxidant effects of aqueous extracts Zanthoxyllum heitzii on sickle cell disorder. BMC Complement Altern Med.

[15] Titus J, Chari S, Gupta M, Parekh N (2004). Pro-oxidant and anti-oxidant status in patients of sickle cell anemia. Indian J Clin Biochem.

[16] Manfredini V, Lazzaretti LL, Griebeler IH, Santin AP, Brandão VDM, Wagner S, Castro SM, Peralba MDCR, Benfato MS (2008). Blood antioxidant parameters in sickle cell anemia patients in steady state. J Natl Med Assoc.

[17] Jain SK, Ross JD, Levy GJ, Duett J (1990). The effect of malonyldialdehyde on viscosity of normal and sickle red blood cells. Biochem Med Metab Biol.

[18] Reiter CD, Gladwin MT (2003). An emerging role for nitric oxide in sickle cell disease vascular homeostasis and therapy. Curr Opin Hematol.

[19] Silva DGH, Edis Belini J, Carrocini CSG, Torres LS, Octávio RJ, Lobo CLC, Domingos CRB, Eduardo AA (2013). Genetic and biochemical markers of hydroxyurea therapeutic response in sickle cell anemia. BMC Med Genet.

[20] Giampiero M, Patrizia C, Pitocco D, Manto A, Mauro AS, Ruotolo V, Caputo S, Giardina B, Ghirlanda G, Santini SA (2002). Early increase of oxidative stress and reduced antioxidant defenses in patients with uncomplicated type 1 diabetes. Diabetes Care.

